# NURSING HOME STAFF CARE APPROACHES ARE ASSOCIATED WITH MEALTIME DIFFICULTIES IN OLDER ADULTS WITH DEMENTIA

**DOI:** 10.1093/geroni/igae098.2692

**Published:** 2024-12-31

**Authors:** Kyuri Lee, Heather Suh, Wen Liu

**Affiliations:** University of Iowa, Iowa City, Iowa, United States; University of Iowa, Iowa City, Iowa, United States; University of Iowa, Iowa City, Iowa, United States

## Abstract

Nonverbal mealtime care is critical for optimizing eating performance and mealtime behaviors of residents with dementia (RwD), especially for those with limited verbal communication abilities. Understanding the relationship between care staff’s nonverbal approaches and RwD’s mealtime behaviors help guide the development of evidence-based, effective mealtime interventions. This observational study characterized staff person-centered and task-centered care approaches and RwD mealtime difficulties and examined the associations between staff care approaches and RwD mealtime difficulties. Videotaped mealtime observations (N=185) involving 47 nursing home direct care staff (age mean ± SD = 45.6±11.8 years; 92.3% female; 80.8% white; 15.5±12.9 years worked as a caregiver, 12.3 ± 9.1 years in the current facility) and 12 RwDs (age 71.9±7.5 years; 91.7% male; 91.7% white, 41.7% married) were coded using the refined Cue Utilization and Engagement in Dementia (CUED) mealtime video coding scheme. Staff primarily used nonverbal person-centered care approaches, including modifications of resident abilities (62.4%), dyadic interactions (10.7%), and the dining environment (6.0%), followed by task-centered behaviors (9.6%). RwD’s mealtime difficulties score assessed by The Edinburgh Feeding Evaluation in Dementia (EdFED) was 5.91±3.59 (range 0-15). RwD’s mealtime difficulties was significantly associated with person-centered care approaches (b=0.04, p=.005) and task-centered care approaches (b=0.11, p=.042), controlling for staff age, years worked as a caregiver, and years worked in the current facility. Minimizing the use of task-centered care may alleviate residents’ mealtime difficulties. Person-centered care is highly recommended in dementia care, but needs to be multifaceted and tailored to the different contexts and needs of individual residents.

